# Absolute and Relative Washout Rates Associated With Parathyroid Adenoma

**DOI:** 10.7759/cureus.48947

**Published:** 2023-11-17

**Authors:** Mohammad Wazzan

**Affiliations:** 1 Department of Radiology, King Abdulaziz University Faculty of Medicine, Jeddah, SAU

**Keywords:** 4d ct, relative washout, parathyroid adenoma, ct scan, absolute washout

## Abstract

Background

Parathyroid adenoma is a benign parathyroid gland tumor that causes excessive parathyroid hormone production, leading to primary hyperparathyroidism. High serum calcium levels characterize it. Accurate diagnosis and localization of adenomas are crucial for effective surgical management. Computed tomography is a fundamental imaging technique used to identify and characterize parathyroid adenomas. This study aimed to provide a comprehensive overview of the absolute and relative contrast washout rates of parathyroid adenoma and the thyroid gland, and compare enhancement patterns to establish the absolute and relative washout rates of parathyroid adenoma.

Materials & methods

This retrospective study analyzes the CT findings of 33 patients with histopathologically proven parathyroid adenomas. All patients with 4D CT scans have been included with no exclusion criteria. The mean attenuation was measured in Hounsfield units for the parathyroid adenoma and thyroid gland in the non-enhancing, arterial, venous, and delayed phases, depending on the region of interest. All statistical analyses were performed using SPSS (IBM Corp., Armonk, NY, USA). Student's t-test was used to evaluate the differences in measurements between the parathyroid adenoma and thyroid tissue. One-way ANOVA was used to evaluate the difference in calculations between the parathyroid adenoma and thyroid tissue. P-values <0.001 were considered statistically significant.

Results

The most common location of parathyroid adenomas is inferior to the thyroid gland. The average pre-contrast attenuation of the parathyroid adenoma is 61.8 ± 15.5 HU compared to 105.5 ± 15.2 HU of the thyroid gland. The arterial attenuation of the parathyroid adenoma is 170.3 ± 40.7 HU, relatively comparable to the thyroid gland arterial attenuation, which is 188.0 ± 9.6 HU. The venous and delayed-phase attenuation of the parathyroid adenoma were 146.8 ± 37.5 and 96.8 ± 26.7 HU, respectively, and 178.8 ± 20.2 HU and 149.3 ± 15.2 HU for the thyroid gland, respectively. The calculated absolute and relative arterial washout rates for the parathyroid adenoma were 69.4 ± 13.4% and 43.2 ± 8.0%, respectively, as compared to 46.4 ± 9.9% and 20.6 ± 6.7% for the thyroid gland. The calculated absolute and relative venous washout rates for the parathyroid adenoma were 58.0 ± 21.4% and 33.0 ± 13.7%, respectively, as compared to 37.2 ± 17.2% and 15.9 ± 9.6% for the thyroid gland.

Conclusions

Parathyroid adenoma demonstrated a significantly higher washout rate than the thyroid gland tissue. Absolute arterial washout ≥69% and relative arterial washout ≥43% indicate parathyroid adenoma. Moreover, absolute venous washout ≥58% and relative venous washout ≥33% can be considered diagnostic factors for parathyroid adenoma. Further, pre-contrast attenuation of <60 Hounsfield units has a substantial predictive value for parathyroid adenoma in addition to the described washout rate. Increased awareness of the washout rate can increase the success rate of four-dimensional computed tomography interpretation.

## Introduction

Parathyroid adenomas are benign neoplasms originating from the parathyroid glands and cause excessive parathyroid hormone production. Over the past decade, ultrasound as the first-line imaging modality and 99mTc sestamibi parathyroid scintigraphy as the second-line imaging modality for parathyroid adenomas has changed considerably. More recently, neck computed tomography (CT) scans have been preferred because they can accurately localize parathyroid adenomas in most patients [1] and provide better anatomic details for surgical planning [2].

The original report on four-dimensional (4D) CT described a distinctive characterization of detailed CT scan imaging [3]. The primary three "dimensions" are axial CT scan images with reformats in coronal and sagittal dimensions, and the enhancement intensity over time is the fourth [3].

Typically, the parathyroid adenoma demonstrates intense enhancement in the arterial phase, followed by contrast washout in the venous and delayed phases. It also shows low attenuation on the pre-contrast images [4]. This behavior was described as a diagnostic pattern for parathyroid adenomas in 1969 by Doppman et al. [5].

The study aimed to describe the CT characteristics of parathyroid adenomas based on CT attenuation and washout rates. Parathyroid 4D CT is a novel imaging tool for detecting parathyroid adenoma. The resolution of 4D CT is superior to that of any other parathyroid scan type [6]. Radiologists have identified abnormal parathyroid adenomas as small as 5 mm, which have been surgically removed, resulting in successful curative parathyroid surgery. This study aimed to elucidate the Hounsfield attenuation and washout rate in the arterial and venous phases when evaluating parathyroid adenomas using 4D CT scans.

## Materials and methods

The author retrospectively reviewed the CT findings of 33 patients with histopathologically proven parathyroid adenomas. All patients who have had 4D CT scans have been included with no exclusion criteria. The mean attenuation was measured in Hounsfield units (HU) for the parathyroid adenoma and thyroid gland in the non-enhancing, arterial, venous, and delayed phases, depending on the region of interest (ROI). The ROI should include at least 2/3 of the target lesion to ensure a representative assessment.

The following washout calculation method was implemented to determine the absolute and relative washout rates for:

$\text{Absolute washout calculation} = \frac{{(\text{HU enhancement phase}) - (\text{HU delayed})}}{{(\text{HU enhancement phase}) - (\text{HU non-enhanced})}} \times 100$

$\text{Relative washout calculation} = \frac{{(\text{HU enhancement phase}) - (\text{HU delayed})}}{{(\text{HU enhancement phase})}} \times 100$

**Figure 1:**
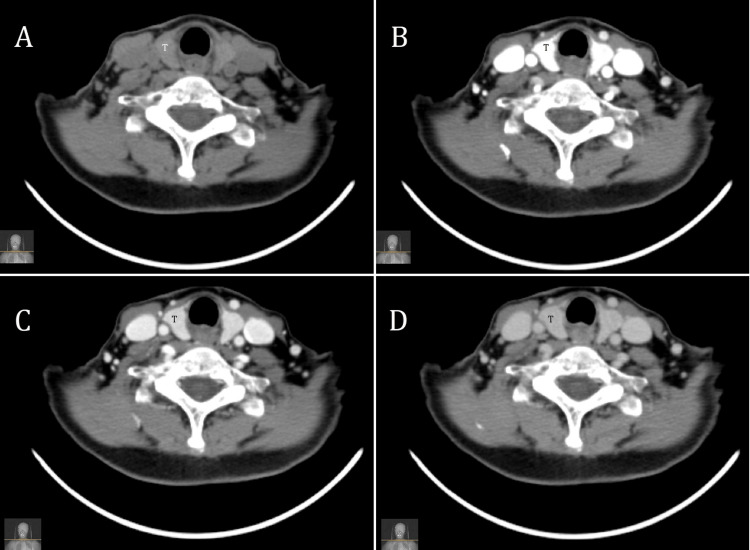
Four-dimensional computed tomography scan of the neck at the level of the thyroid gland. This is an axial CT scan of the neck at the level of the thyroid gland (T) for a 32-year-old female patient in four different phases; Pre-contrast phase (A), arterial phase (B), venous phase (C), and delayed phase (D).

The CT scans were performed for all patients using one of three CT scanners, ranging from a 32-slice to a 128-slice multidetector computed tomography Siemens scanner (Siemens Healthineers, Erlangen, Germany). The imaging technique consists of four scans (Figure 1). The first was a non-enhanced CT study. The subsequent three scans were contrast-enhanced. The arterial phase was acquired 25 seconds after injection. The venous phase was obtained 80 seconds from infusion initiation, and the delayed phase was acquired 300 seconds from injection. For all scans, the section thickness was 0.625 mm, the kVp was 140, the exposure time was 250 ms, and the field of view was 350 mm. The CT images were acquired in the craniocaudal direction during a single breath-hold for all examinations. The images were transferred to the patient archiving and communication system for interpretation.

Statistical analysis

All statistical analyses were performed using SPSS version 23.0 software (IBM Corp., Armonk, NY, USA). Student's t-test was used to evaluate the differences in measurements between the parathyroid adenoma and thyroid tissue. One-way ANOVA was used to evaluate the difference in calculations between the parathyroid adenoma and thyroid tissue. P-values <0.001 were considered statistically significant.

## Results

The most common location of parathyroid adenomas was inferior to the thyroid gland (75%), with 39% on the left side and 36% on the right side (Table 1). The average size of the parathyroid adenoma was 10.6 ± 3.7 mm. The smallest adenoma measured 6 mm in diameter, and the largest was 22 mm in diameter.

**Table 1:** Distribution of parathyroid adenoma in relation to the thyroid gland The table summarizes the most common location of the parathyroid adenoma in relation to the thyroid gland for the sample size (N).

Adenoma location	N (33) * (%)
Left and Inferior	13 (39%)
Right and Inferior	12 (36%)
Right and Superior	3 (9%)
Left and medial	3 (9%)
Left and Superior	2 (6%)

The average pre-contrast attenuation of the parathyroid adenoma is 61.8 ± 15.5 HU compared to 105.5 ± 15.2 HU of the thyroid gland. The arterial attenuation of the parathyroid adenoma is 170.3 ± 40.7 HU, relatively comparable to the thyroid gland arterial attenuation, which is 188.0 ± 9.6 HU. The venous and delayed-phase attenuation of the parathyroid adenoma were 146.8 ± 37.5 and 96.8 ± 26.7 HU, respectively, and 178.8 ± 20.2 HU and 149.3 ± 15.2 HU for the thyroid gland, respectively (Table 2).

**Table 2:** Parathyroid adenoma and thyroid tissue Hounsfield measurements in the pre-contrast, arterial, venous, and delayed phases of 4D CT scan The table represents the minimum, maximum, and mean attenuation of the parathyroid adenoma as compared to the thyroid tissue in the pre-contrast, arterial, venous, and delayed phases. Note the significantly higher attenuation of the thyroid gland tissue as compared to the parathyroid adenoma. Also, note the slow contrast washout of the thyroid tissue as compared to the parathyroid adenoma. P-values <0.001 were considered statistically significant. CT, computed tomography; HU, Hounsfield units; SD, standard deviation; 4D, four-dimensional.

CT enhancement phase	Parathyroid adenoma attenuation		Thyroid tissue attenuation		p-value
	Min–Max	Mean ± SD (HU)	Min–Max	Mean ± SD (HU)	
Pre-contrast attenuation	29.0–86.0	61.8 ± 15.8 HU	72.0–130.0	105.5 ± 15.2 HU	<0.001
Arterial phase enhancement	116.0–260.0	170.3 ± 40.7 HU	160.0–205.1	188.0 ± 9.6 HU	0.025
Venous phase enhancement	95.0–219.3	146.8 ± 37.5 HU	152.0–253.0	178.8 ± 20.2 HU	<0.001
Delayed phase enhancement	49.0–156.0	96.8 ± 26.7 HU	110.0–165.9	149.3 ± 15.2 HU	<0.001

The following arterial washout calculation equations were implemented to determine the absolute and relative washout rates:

$\text{Absolute arterial washout calculation} = \frac{{(\text{HU arterial}) - (\text{HU delayed})}}{{(\text{HU arterial phase}) - (\text{HU non-enhanced})}} \times 100$

$\text{Relative arterial washout calculation} = \frac{{(\text{HU arterial phase}) - (\text{HU delayed})}}{{(\text{HU arterial phase})}} \times 100$

The calculated absolute and relative arterial washout rates for the parathyroid adenoma were 69.4 ± 13.4% and 43.2 ± 8.0%, respectively, as compared to 46.4 ± 9.9% and 20.6 ± 6.7% for the thyroid gland.

For the venous washout calculation, the following equations were implemented:

$\text{Absolute venous washout calculation} = \frac{{(\text{HU venous}) - (\text{HU delayed})}}{{(\text{HU venous phase}) - (\text{HU non-enhanced})}} \times 100$

$\text{Relative venous washout calculation} = \frac{{(\text{HU venous phase}) - (\text{HU delayed})}}{{(\text{HU venous phase})}} \times 100$

The calculated absolute and relative venous washout rates for the parathyroid adenoma were 58.0 ± 21.4% and 33.0 ± 13.7%, respectively, as compared to 37.2 ± 17.2% and 15.9 ± 9.6% for the thyroid gland (Table 3).

**Table 3:** Absolute and relative arterial and venous washout of the parathyroid adenoma and thyroid gland The table represents the minimum, maximum, and mean absolute and relative washout rates of the parathyroid adenoma as compared to the thyroid tissue. Note the significantly higher washout rates for the parathyroid adenoma as compared to the thyroid gland. P-values <0.001 were considered statistically significant. SD, standard deviation.

Washout rate	Parathyroid adenoma washout rate		Thyroid tissue washout rate		p-value
	Min–Max	Mean ± SD (%)	Min–Max	Mean ± SD (%)	
Absolute arterial washout	39.7–93.5	69.4 ± 13.4%	28.6–71.9	46.4 ± 9.9%	<0.001
Relative arterial washout	23.5–58.3	43.2 ± 8.0%	8.8–36.4	20.6 ± 6.7%	<0.001
Absolute venous washout	20.0–93.4	58.0 ± 21.4%	4.1–87.0	37.2 ± 17.2%	<0.001
Relative venous washout	11.2–55.6	33.0 ± 13.7%	1.2–42.3	15.9 ± 9.6%	<0.001

## Discussion

Primary hyperparathyroidism is primarily caused by parathyroid adenomas, and accurate localization of these adenomas is essential for surgical intervention [7]. The enhanced diagnostic capabilities of 4D CT can lead to improved surgical planning and outcomes. Clinicians can confidently identify the presence of a parathyroid adenoma and select the appropriate surgical approach (e.g., minimally invasive parathyroidectomy) [8]. This results in more effective and precise treatment of primary hyperparathyroidism, reducing the risk of recurrence.

The high resolution and sensitivity of 4D CT enable the detection of smaller parathyroid adenomas. This precise detection can lead to curative surgical procedures, preventing the progression of primary hyperparathyroidism and its associated complications. Patients can benefit from timely intervention, including minimally invasive surgery, which results in smaller incisions, reduced scarring, and faster recovery [8].

In cases where other imaging modalities, such as ultrasound and scintigraphy, fail to localize the adenoma, 4D CT can be a valuable adjunct. Its ability to provide detailed anatomical information, precise localization, and quantitative measurements of contrast enhancement and washout makes it a powerful tool in challenging and complex cases. This helps clinicians avoid unnecessary exploratory surgery and ensures that patients receive the most appropriate treatment [8].

The study's findings highlight the diagnostic potential of 4D CT for parathyroid adenomas. The use of attenuation measurements and washout rates is critical for differentiating these adenomas from surrounding tissues, especially the thyroid gland. The proposed diagnostic criteria, such as absolute and relative arterial and venous washout rates, provide objective and quantifiable parameters for identifying parathyroid adenomas. The study's thresholds of washout rates (≥69% for arterial and ≥43% for relative arterial, ≥58% for venous, and ≥33% for relative venous) can serve as a practical guideline for clinicians. These criteria offer a more standardized and precise approach to diagnosis, reducing the subjectivity of interpretation.

Parathyroid adenomas vary in size, ranging from a few millimeters to a few centimeters. The most common location was inferior to the thyroid gland, which is supported by the findings of many previous studies [4]. They can also occur in ectopic locations, such as the mediastinum or retroesophageal region. Ectopic parathyroid adenomas account for 20-25% of all cases [9,10].

**Figure 2:**
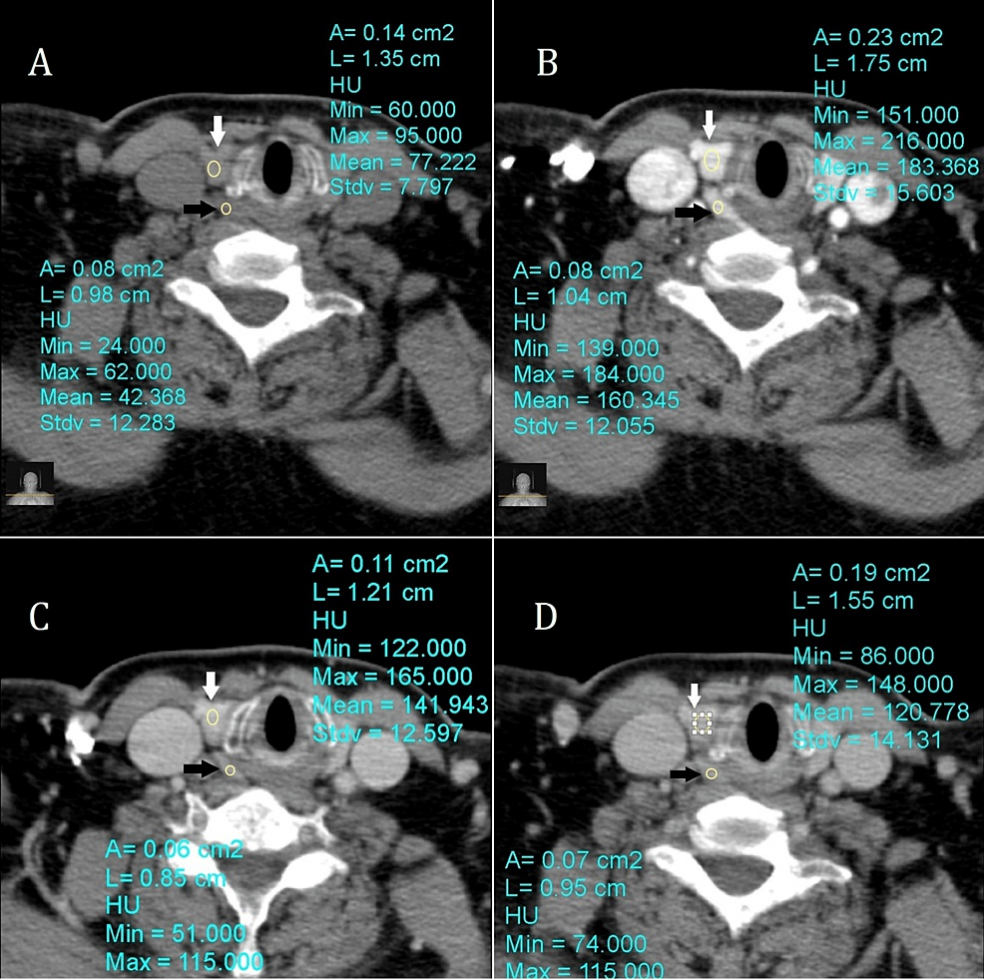
Demonstration of measurement technique on the axial images of the 4-dimensional CT scan. Axial images on the non-enhanced (A), arterial (B), venous (C), and delayed (D) phases at the level of the thyroid gland. In the precontrast scan (A), note the significantly higher attenuation measured by ROI (yellow circle) of the thyroid gland (white arrow) as compared to the parathyroid adenoma (black arrow). The parathyroid adenoma (black arrow) as well as the thyroid gland (white arrow) demonstrate the highest attenuation in the arterial phase (B). In the venous (C) and delayed (D) phases, note the rapid washout of the contrast from the parathyroid adenoma (black arrow).

On non-contrast CT images, adenomas typically appear as well-defined soft tissue attenuated masses with a lower density than the adjacent thyroid tissue. The primary role of the non-enhanced phase was to differentiate a parathyroid adenoma from high-attenuation iodine-containing thyroid tissue (Figure 2) and to calculate the absolute washout rate [4].

The parathyroid adenoma demonstrates maximum attenuation in the arterial phase, ranging from 116 to 260 HU [11,12], due to intense contrast enhancement. Therefore, it is recommended to start interpretation by the arterial phase. Classically, parathyroid adenomas demonstrate higher attenuation than the thyroid in the arterial phase and lower attenuation than the thyroid in the venous phase. However, this classic enhancement is noted in 20% of adenomas. In addition, parathyroid adenomas may also contain cystic, fat, or calcific components [13].

When a suspected parathyroid adenoma is identified, correlation with the non-enhanced and delayed phase should be performed for the enhancement patterns and washout rates. The characteristic enhancement of a parathyroid adenoma is intense arterial enhancement prior to washout in the venous and delayed phases and lower attenuation than the thyroid gland in the non-enhanced phase.

Lymph nodes are the main mimics of parathyroid adenomas in terms of size. However, lymph nodes show progressive contrast uptake, with peak enhancement in the delayed phase [11,12]. The thyroid tissue can be enhanced intensely in the arterial phase, mimicking the arterial enhancement of parathyroid adenoma. However, it will also have a much slower contrast washout in the delayed phases [11,12]. Moreover, due to the presence of iodine, the thyroid tissue demonstrates significantly high attenuation in the pre-contrast scan (Figure 2).

This study had some limitations. First, it had a limited sample size, which widened the range of the washout rates. Second, it is retrospective in nature. Moreover, this study was only performed at our institution if sonography and nuclear medicine failed to localize the parathyroid adenoma. Future validation of this method requires prospective studies with a larger sample size.

In summary, the study's findings in characterizing parathyroid adenomas have significant implications for the management of primary hyperparathyroidism. This technique offers a more precise and objective approach to diagnosis and localization, potentially improving patient outcomes and reducing the need for exploratory surgery. Further research, validation of diagnostic criteria, and advancements in imaging technology are promising avenues for enhancing the role of 4D CT in diagnosing and managing parathyroid adenomas.

## Conclusions

The study emphasizes the transformation of parathyroid adenoma diagnosis by establishing objective diagnostic criteria and provides a comprehensive understanding of the absolute and relative washout rates associated with parathyroid adenomas, offering radiologists a more accurate and standardized approach to diagnosis. Parathyroid adenoma demonstrated a significantly higher washout rate than the thyroid gland tissue. Absolute arterial washout ≥69% and relative arterial washout ≥43% indicate parathyroid adenoma. Furthermore, absolute venous washout ≥58% and relative venous washout ≥33% can be considered diagnostic factors for parathyroid adenoma. While acknowledging the study's limitations, these findings highlight the importance of further research and validation to strengthen the diagnostic washout rates criteria and the potential for advanced imaging technology in the field.
